# More than just trash bins? Potential roles for extracellular vesicles in the vertical and horizontal transmission of yeast prions

**DOI:** 10.1007/s00294-015-0534-6

**Published:** 2015-11-09

**Authors:** Mehdi Kabani, Ronald Melki

**Affiliations:** Centre National de la Recherche Scientifique (CNRS), Paris-Saclay Institute of Neuroscience, Université Paris-Saclay, Bât. 32-33, Avenue de la Terrasse, 91190 Gif-sur-Yvette, France

**Keywords:** Yeast, Prion, SUP35, [*PSI*^+^], Extracellular vesicle, Exosome

## Abstract

In the yeast *Saccharomyces cerevisiae*, an ensemble of structurally and functionally diverse cytoplasmic proteins has the ability to form self-perpetuating protein aggregates (e.g. prions) which are the vectors of heritable non-Mendelian phenotypic traits. Whether harboring these prions is deleterious—akin to mammalian degenerative disorders—or beneficial—as epigenetic modifiers of gene expression—for yeasts has been intensely debated and strong arguments were made in support of both views. We recently reported that the yeast prion protein Sup35p is exported via extracellular vesicles (EV), both in its soluble and aggregated infectious states. Herein, we discuss the possible implications of this observation and propose several hypotheses regarding the roles of EV in both vertical and horizontal propagation of ‘good’ and ‘bad’ yeast prions.

## Background

**Fig. 1:**
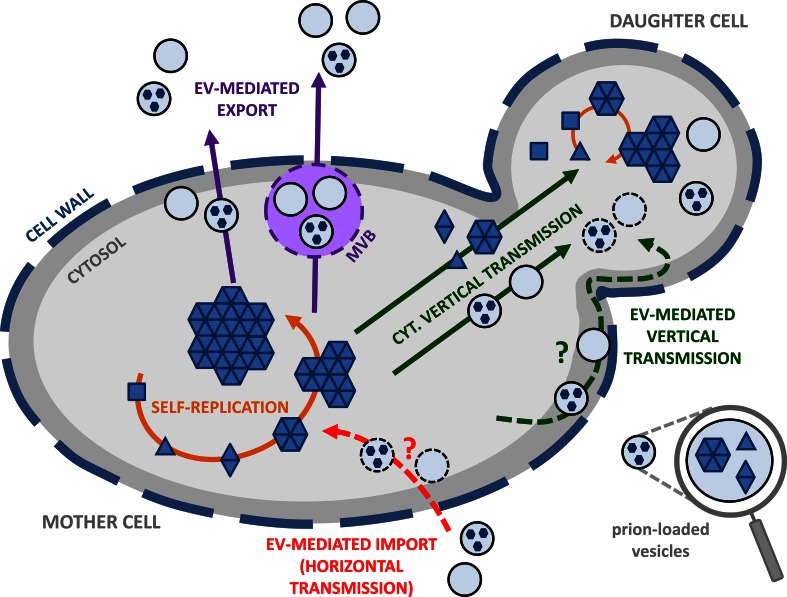
Potential roles for extracellular vesicles in vertical and horizontal yeast prion propagation. Yeast prions self-replicate by the conversion of newly synthesized soluble monomers (*squares*) to a prion conformation (*triangles*) and their subsequent incorporation within high-molecular weight prion assemblies (*solid orange arrows*). Unidentified prion species or ‘propagons’ are vertically and cytoplasmically transmitted to the daughter cell (or to the mating partner) (*solid green arrows*) where they initiate a new round of prion self-replication. Yeast prions are packaged within vesicles which are exported across the cell wall (depicted as a *thick dashed line* to highlight its dynamic nature) and to the extracellular medium (*solid purple arrows*). It remains to be determined whether this process occurs directly and/or via the multi-vesicular bodies (MVB) pathway. Cell-to-cell horizontal transmission of yeast prions may occur if extracellular vesicles are able to cross the cell wall barrier under specific physiological or environmental conditions (*dashed red arrow*). The content of these vesicles may then be released within the cytosol and initiate prion self-replication. Vertical transmission of yeast prions could also be mediated by propagons packaged within intracellular (*solid green arrow*) or extracellular (*dashed green arrow*) vesicles transported from mother to daughter cells where their content would be released in the cytosol to initiate a new cycle of prion self-replication

“Proteinaceous infectious particles”, or prions, are self-perpetuating alternate conformations of proteins that manifest as dominant and cytoplasmically inherited epigenetic traits in mammals, filamentous fungi and yeasts (Aigle and Lacroute 1975; Coustou et al. 1997; Cox 1965; Prusiner 1982; Wickner 1994). The yeast *Saccharomyces cerevisiae* hosts many prion or prion-like proteins, unrelated in sequence and function, among which Sup35p, Ure2p and Rnq1p which cause the [*PSI* ^+^], [*URE3*] and [*PIN* ^+^] traits, respectively. Prions are thought to self-replicate by a nucleated polymerization mechanism which can be recapitulated in vitro with purified recombinant proteins (Kabani et al. 2011; Liebman and Chernoff 2012). Newly synthesized monomers are converted to the prion conformation and continuously recruited within existing prion aggregates (Fig. 1, *self*-*replication*, solid orange arrows) (Kabani et al. 2011; Liebman and Chernoff 2012). As prion proteins populate large ensembles of conformations, they can generate distinct strains which exhibit different seeding propensities resulting in diverse phenotypic manifestation forms (Bradley et al. 2002; Bradley and Liebman 2003; Derkatch et al. 1996). These high-molecular prion assemblies are dynamic and are remodeled by molecular chaperones (Kabani et al. 2011; Liebman and Chernoff 2012). Prions are faithfully transmitted to daughter cells (or to mating partners) via cytosolic diffusible protein entities often referred to as ‘propagons’, the exact molecular nature of which is currently not known (Fig. 1, *cytosolic vertical transmission*, solid green arrows).

We have recently showed that infectious Sup35p prion particles are packaged within small exosome-like vesicles (~30–100 nm in diameter) and exported in the extracellular space (Fig. 1, *EV*-*mediated export*, solid purples arrows) (Kabani and Melki 2015). These extracellular vesicles (EV) are produced by virtually all cells from the three domains of life—*Bacteria, Archaea, Eukarya*—including yeasts and fungi. EV are thought to play important roles for intercellular communication by acting as vehicles for the transfer of nucleic acids, proteins, signaling molecules, sugars, lipids, and toxins or other pathogenic factors. EV may also mediate the clearance and possibly the cell-to-cell transfer of prion-like protein aggregates associated with neurodegenerative diseases (Albuquerque et al. 2008; Oliveira et al. 2010; Peres da Silva et al. 2015; Rajendran et al. 2014; Rodrigues et al. 2007, 2008; Simons and Raposo 2009).

While we certainly need to establish whether prions other than Sup35p are exported via EV, we believe our findings have important implications and would here like to propose several hypotheses as to the potential role of EV in yeast prion clearance and/or propagation.

## Yeast prions: friends or foes?

In trying to understand the implications of EV-mediated export of yeast prions, we need to consider that the latter have been proposed to have both detrimental and beneficial effects on yeast cells and populations. Extensive debate over this issue falls outside the scope of the present perspective review, but we will briefly summarize below some of the main arguments in favor of these mutually non-exclusive hypotheses.

Yeast prions were long considered as deleterious abnormal misfolded protein aggregates as (1) the phenotypes associated with the prion state often mimics that of a loss-of-function allele which can cause major cellular dysfunctions; (2) the levels of molecular chaperones are often elevated in prion-containing cells indicating these resent prions as a stress; (3) mammalian prions, prion variants or prion-like proteins were reported to be cytotoxic or lethal (Liebman and Chernoff 2012; Tuite 2015; Wickner et al. 2011, 2015).

Yeast prions have also been described as beneficial under defined circumstances (Garcia and Jarosz 2014; Halfmann et al. 2010; Newby and Lindquist 2013; Shorter and Lindquist 2005) as: (1) they are rather well tolerated and are very stable in many laboratory strains; (2) they do not confer selective disadvantages as those studied in laboratory conditions were also found in wild yeasts from diverse ecological niches; (3) they have been proposed, because of their involvement in transcriptional and translational control, to act as epigenetic modifiers of gene expression allowing adaptation and survival under challenging growth conditions; (4) functional amyloids exist in yeast and filamentous fungi where they play diverse roles in the cell cycle, gametogenesis or biofilm formation, and in higher eukaryotes.

Truth most probably lies somewhere between these two extreme views: some prions or prion variants may be toxic or even lethal, while others may be advantageous (or neutral) under defined circumstances. Hence, in the following paragraphs, we will propose and discuss several hypotheses regarding the roles of EV in the propagation of yeast prions, taking into account that these could be either deleterious (i.e., something that needs to be eliminated) or advantageous (i.e, something that needs to be maintained and transmitted).

## EV-mediated prion export as a quality control mechanism?

EV-mediated export of yeast prions (Fig. 1, *EV*-*mediated export*, solid purples arrows) contributes to the dilution of infectious misfolded protein aggregates. This process may therefore relieve the cell from potentially harmful protein aggregates. Consistent with this view is the observation that higher levels of Sup35p are present within EV recovered from [*PSI* ^+^] as compared to those from [*psi* ^−^] cells (Kabani and Melki 2015). A triage process allowing the preferential packaging of aggregated prions into EV could also account for that of soluble misfolded non-functional or ‘aged’ Sup35p molecules that were resilient to proteolytic elimination (Kabani et al. 2014). While this needs to be demonstrated and established as a generic process, the export of misfolded or aggregated polypeptides would contribute to cells well-being by lowering the burden on the cellular quality control machineries (i.e. molecular chaperones, ubiquitin–proteasome system, etc.). As the amount of Sup35p found within EV is low compared to that in the cytosol, in both [*psi* ^−^] or [*PSI* ^+^] cells (Kabani and Melki 2015), an EV-mediated clearance mechanism would most likely play a secondary role in maintaining cellular proteostasis.

## EV-mediated vertical transmission of propagons?

The cytoplasmic transmission of yeast prions during cell division is well established yet the molecular nature of the propagons still remains elusive (Fig. 1; *vertical transmission,* solid green arrows). Prion particles encompass a wide variety of sizes, from small to very large assemblies which can be visualized by differential centrifugation and under the microscope using for instance GFP-tagged prions (Kabani and Melki 2011; Liebman and Chernoff 2012). The core-aggregates that are believed to represent the building bricks of these assemblies can also be visualized on agarose gels under semi-denaturant conditions (Kryndushkin et al. 2003). However, it is unclear whether these high molecular weight species and *bona fide* propagons correspond to the same entities (e.g. Dulle et al. 2013). It is also not clear whether the propagons are naked, coated with associated proteins or encapsulated into vesicles (Kabani and Melki 2015). Propagons are expected to meet a number of requirements: (1) they must contain the structural information needed for faithful seeding of monomeric prions; (2) their size and mobility must allow them to traffic across the bud neck to the daughter cell. This active or passive transport needs to be very efficient as most prions are mitotically stable under laboratory conditions; (3) they must show a significant level of resistance toward the action of protein assemblies remodeling activities within the cell, e.g. molecular chaperones and degradation machineries (Kabani et al. 2014; Wang et al. 2015).

It has been known for a long time that secretory vesicles significantly contribute to the growth of the bud in *S. cerevisiae* where they provide the macromolecules and enzymes required for building the newly forming plasma membrane and cell wall of the daughter cell (Novick and Schekman 1979). Similarly, EV were also proposed to deliver cell wall constituents and remodeling enzymes to the periplasmic space (Brown et al. 2015). Therefore, it is tempting to speculate that prion-containing vesicles could very well traffic from mother to daughter cells—either directly through the cytosol (Fig. 1, *cytosolic vertical transmission,* solid green arrow) or after export in the periplasmic space (Fig. 1, *EV*-*mediated vertical transmission*, dashed green arrow)—where their content would be released allowing a new cycle of prion self-replication to begin (Fig. 1, *daughter cell*, solid orange arrow). By hijacking the mother-to-bud vesicular traffic, propagons could escape remodeling or proteolytic activities within the cell and circumvent possible asymmetric retention within mother cells, a mechanism that concurs to aging of yeast mother cells and to the rejuvenation of daughter cells (Liu et al. 2010; Tessarz et al. 2009; Zhou et al. 2011).

## EV-mediated horizontal cell-to-cell transfer of propagons?

Finally, the most intriguing implication of our observations is the potential existence of a horizontal cell-to-cell transfer of yeast prions (Kabani and Melki 2015). The proof-of-principle that exogenous prion particles can induce the prion state if they are introduced in naïve cells has already been made over a decade ago (Brachmann et al. 2005; King and Diaz-Avalos 2004; Patel and Liebman 2007; Tanaka et al. 2004). We showed that EV isolated from [*PSI* ^+^] cells are infectious (Kabani and Melki 2015). This means that infectivity is not lost upon encapsulation of Sup35p propagons within EV. It is still unknown whether yeast cells can naturally capture such entities from the extracellular medium and transport them across the cell wall (Fig. 1, *EV*-*mediated import, horizontal transmission*, dashed red arrow). The mechanisms by which vesicles cross the cell wall following physiological and environmental changes within the latter are also not known but several non-mutually exclusive hypotheses have been made (Brown et al. 2015; Wolf and Casadevall 2014). The vesicles could be forced through the cell wall by turgor pressure after release in the periplasmic space; cell wall-remodeling enzymes, associated or not to the vesicles, could facilitate the passage of the vesicles by locally loosening the polysaccharides (chitin/β-glucan/mannan) mesh; vesicles could also transit through cell wall-spanning channels with the help of cytoskeleton-associated structural cables (Wolf and Casadevall 2014). We envision EV-import as a three-step event mirroring EV-export: (1) EV could first bind to yeast cells (with or without the help of EV surface-associated proteins); (2) EV-associated cell wall-modifying enzymes (Albuquerque et al. 2008) could facilitate EV access to the plasma membrane; (3) and EV could be subsequently internalized by endocytosis in response to specific cues such as glucose concentration in the medium (Giardina et al. 2014).

## EV-mediated, prion-based cell-to-cell communication?

In the wild, microorganisms are found in complex communities or biofilms where they cooperate or compete for resources. Interestingly, a cross-kingdom communication has been described where bacteria produce a diffusible chemical factor that induces a specific prion ([*GAR* ^+^]) in wild yeasts. This has been described as a mutually beneficial event since [*GAR* ^+^] yeast cells are able to use multiple carbon sources even in the presence of glucose but in turn produce less ethanol which favors the growth and viability of the bacteria (Jarosz et al. 2014). Thus, it may be that the environmental growth conditions and the complex interactions among microbes, besides influencing the formation, transmission and elimination of yeast and fungal prions, use the latter as messengers or sensors. Indeed, quorum sensing mechanisms in bacteria and fungi allow the coordination of cell physiology among individual cells according to environmental growth conditions and/or surrounding (micro)organisms (Albuquerque and Casadevall 2012; Chen and Fink 2006; Leonhardt et al. 2015; Waters and Bassler 2005; Yang and Lan 2015). This cell-to-cell communication is generally mediated by hormone-like small diffusible chemical factors produced by these microorganisms and to which recipient cells respond by reprogramming their transcriptome, proteome and/or metabolome. If prion-trafficking between neighbor cells of the same or distinct species via EV is demonstrated in the future, we anticipate that their ability to modulate gene expression makes them good candidates for a new and sensitive type of protein-based quorum sensing mechanism.

What would be the purpose of horizontal transfer of fungal prions? Advocates of the ‘prions are mostly bad’ view would think of it as a pathogenic mechanism destined to disseminate prions among different fungal and yeast species. The existence of species barriers that prevent cross-species prion infection could then be viewed as a defensive mechanism against such a threat. Advocates of the ‘prions can be beneficial’ view would rather think of it as a way to maintain and disseminate an evolutionary-advantageous factor among closely related species. In this context, cross-species prion barriers would allow this selective advantage to be kept within a group of distinct species.

## Conclusions

That thick-walled microorganisms such as gram-positive bacteria, yeasts and fungi are able to secrete small vesicles in the extracellular space was only recently confirmed (Brown et al. 2015). These EV contain a wide range of cargo including lipids, sugars, nucleic acids, proteins and toxins and their roles in microbial physiology, communication and pathogenicity are only beginning to emerge (Brown et al. 2015). We believe our observation that a yeast prion is exported in these EV to be important and will allow us to ask and solve new issues in both the prion and EV fields (Kabani and Melki 2015). Understanding how and when prions (or other protein aggregates) are selected and packaged inside vesicles, and how these vesicles traffic across the cell wall and between yeast cells is of an interest that surpasses the yeast prion field. Indeed, misfolded protein assemblies which are the causative agents of many degenerative diseases such as Alzheimer’s or Parkinson’s diseases have been found in EV which have been proposed to act as vehicles for the prion-like spreading pattern of these diseases (Rajendran et al. 2014). We hope that our initial work (Kabani and Melki 2015) and the elements discussed in this perspective will stimulate more work on this exciting new area of research.
